# Co-occurrence of *VHL* and *SDHA* Pathogenic Variants: A Case Report

**DOI:** 10.3389/fonc.2022.925582

**Published:** 2022-07-07

**Authors:** Moon Ley Tung, Bharatendu Chandra, Kyle Dillahunt, Matthew D. Gosse, T. Shawn Sato, Alpa Sidhu

**Affiliations:** ^1^ Division of Medical Genetics and Genomics, The Stead Family Department of Pediatrics, University of Iowa Hospitals and Clinics, Iowa City, IA, United States; ^2^ Department of Pathology, University of Iowa Hospitals and Clinics, Iowa City, IA, United States; ^3^ Division of Pediatric Radiology, The Stead Family Children’s Hospital, University of Iowa Hospitals and Clinics, Iowa City, IA, United States

**Keywords:** von Hippel-Lindau syndrome, *SDHA*-associated paraganglioma and pheochromocytoma syndrome, paraganglioma, genetics, cancer

## Abstract

Von Hippel Lindau(VHL)syndrome presents with cerebellar and spinal hemangioblastomas, renal cell cancer, neuroendocrine pancreatic tumor, and pheochromocytoma and it is caused by germline mutations in the *VHL* gene. Pathogenic germline variants in the succinate dehydrogenase A (*SDHA*) gene are associated with paraganglioma and pheochromocytoma. Here we report co-occurrence of germline pathogenic variants in both *VHL* and *SDHA* genes in a patient who presented with pancreatic neuroendocrine tumor. As these genes converge on the pseudo-hypoxia signaling pathway, further studies are warranted to determine the significance of co-occurrence of these variants in relation to tumor penetrance, disease severity, treatment response and clinical outcomes in this selected group of patients.

## Introduction

Von Hippel Lindau (VHL) syndrome caused by germline loss-of-function variants in the gene *VHL*, is an autosomal dominant cancer predisposition syndrome (1). Clinical features include cerebellar and spinal hemangioblastomas, renal cell cancer, neuroendocrine pancreatic tumor, pheochromocytoma, and paragangliomas. Germline pathogenic variants in the succinate dehydrogenase A (*SDHA*) gene are associated with familial paraganglioma and pheochromocytoma syndrome inherited in an autosomal dominant manner (2). At the cellular level, SDHA and VHL proteins interact and converge into a common molecular pathway *via* the hypoxia inducible factor alpha (HIF-α) (3). Here we report co-occurrence of germline pathogenic variants in both genes (*VHL* and *SDHA*) in a patient who presented with pancreatic neuroendocrine tumor. We describe the known molecular pathways involving VHL and SDHA and postulate that the disease prognosis may be dependent on the presence of co-occurring pathogenic variants in these genes through the involvement of HIF-α in the final common pathway. To our knowledge, this is the first case report of both *VHL* and *SDHA* pathogenic variants.

## Case Presentation

**Figure 1:**
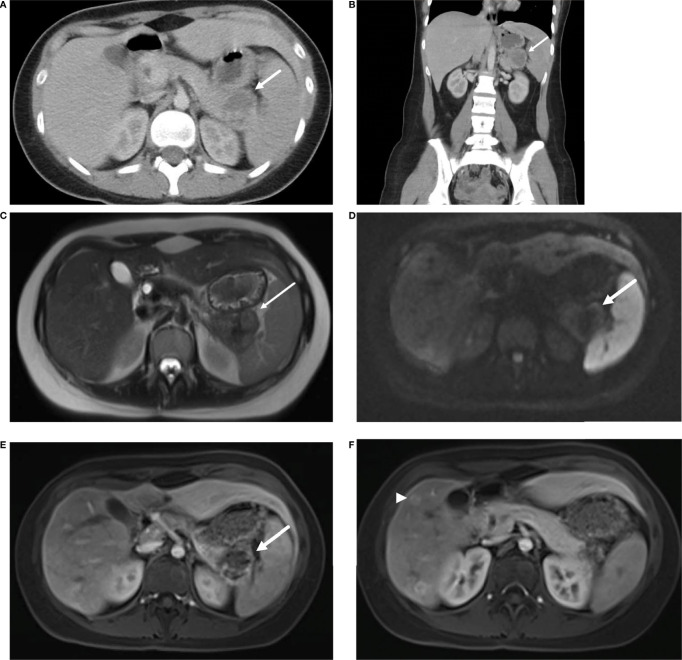
Axial **(A)** and coronal **(B)** contrast enhanced CT demonstrate a pancreatic tail mass with central necrosis (→) positioned between the stomach, spleen, and kidney. MRI of the abdomen reveals a mass in the tail of the pancreas (→) with intermediate T2 signal **(C)**, diffusion restriction **(D)** and peripheral contrast enhancement **(E)**. Additional enhancing lesions (▸) were seen in the liver consistent with metastatic disease **(F)**.

The proband, a 23-year-old female, was reviewed at our Genetics Cancer Predisposition clinic for evaluation and recommendations for concurrent germline pathogenic variants in the *VHL* and *SDHA* genes. She presented with recurrent episodes of abdominal pain, vomiting, and anorexia at 21-years of age. Initial radiological and endoscopic evaluations were unremarkable. Her symptoms continued to persist, and a review of her prior abdominal computed tomography (**Figures 1A, B**) and magnetic resonance imaging (MRI) study showed a mass in the pancreatic tail with hepatic metastases (**Figures 1C–F**). She underwent surgical resection of pancreatic tail mass as well as right liver lobectomy, splenectomy, and cholecystectomy for metastatic disease.

**Figure 2:**
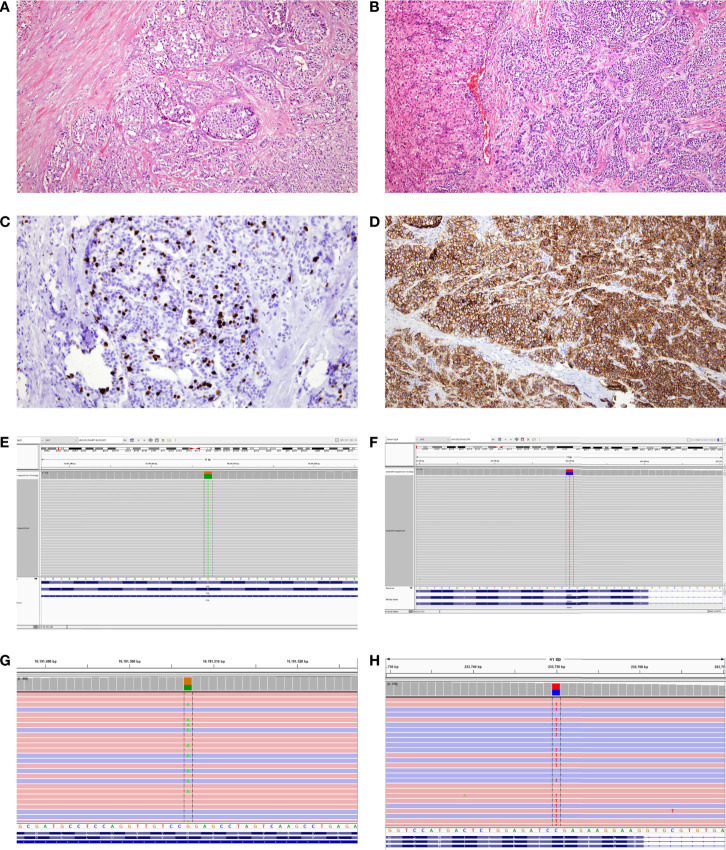
**(A)** Hemotoxylin and eosin (H&E) staining of primary tumor. Cells are arranged in nests with finely stippled chromatin. The tumor cells expressed synaptophysin, chromogranin, and pankeratin (not shown) supporting the diagnosis of well-differentiated neuroendocrine tumor. **(B)** H&E stained section of metastatic tumor focus in the liver with similar morphology to the primary tumor. **(C)** Ki-67 immunostained section of the metastatic tumor focus in the liver. The proliferation index was found to be 11% in a similar hot spot by digital image analysis. **(D)** Somatostatin receptor subtype 2A (SSTR2A) immunostained section was positive (3+, 90%). **(E)** Tumor molecular profiling showing a variant in *VHL* (c.500G>A, p.R167Q) at a frequency of 66.3%). **(F)** Tumor molecular profiling showing a variant in *SDHA* (c.1054C>T, p.R352*) at a frequency of 50%). **(G)** Germline molecular profiling showing an identical variant in *VHL* (c.500G>A, p.R167Q). **(H)** Germline molecular profiling showing an identical variant in *SDHA* (c.1054C>T, p.R352*).

Histopathologic examination of the pancreatic tail mass revealed a well-differentiated neuroendocrine tumor (**Figure 2A**). The primary tumor was centered in the distal pancreas, perineural invasion was present, and 4 mitoses per 2 mm^2^ were identified. Metastatic tumor deposits were identified in two of fifteen regional lymph nodes and multiple metastatic liver foci were identified (**Figure 2B**). A Ki-67 immunostain was performed on the primary tumor, local lymph node metastasis, and liver metastasis (**Figure 2C**) and the proliferation index was determined to be 14%, 8.5%, and 11%, respectively, using digital image analysis. This met criteria for a Grade 2 (intermediate) tumor by 2019 WHO classification using both mitotic count and Ki-67 proliferation index. Immunohistochemistry on the primary tumor was positive for synaptophysin, chromogranin, pan keratin, and CK7. and negative for CK20, CD10 and progesterone receptor (PR). Somatostatin receptor subtype 2A (SSTR2A) immunostaining was positive (3+, 90%) (**Figure 2D**) and ATRX immunostain was intact. Initial testing of tumor was performed at an outside institution and additional pathology samples were not available for SDHB immunohistochemistry.

Molecular test performed on the tumor sample showed pathogenic variants in the *VHL* gene (c.500G>A, p.R167Q) (**Figure 2E**) involving 66% of tumor cells, and *SDHA* gene (c.1054C>T, p.R352*) (**Figure 2F**) involving 50% of tumor cells. Germline testing on peripheral blood sample showed identical pathogenic variants in *VHL* (c.500G>A, p.R167Q) (**Figure 2G**) and *SDHA* (c.1054C>T, p.R352*) genes (**Figure 2H**). Familial testing could not be performed due to patient’s adoptive status. A Positron Emission Tomography scan following the surgery was suggestive of multiple somatostatin receptor tracer uptake in the pancreatic head, duodenum, and the liver. She was initially treated with octreotide acetate and subsequently with Lantreotide.

The patient’s past medical history was significant for a right retinal hemangioma diagnosed at 18- years of age. Follow-up ophthalmological evaluation showed consistent exam without progression of disease. She was making good recovery from her initial diagnosis at the time of our review, and the pancreatic and hepatic lesions have remained stable. She was recommended studies per surveillance guidelines for her diagnosis of both *VHL* and *SDHA*-related paraganglioma and pheochromocytoma syndrome. She had normal brain, internal auditory canal, and spine magnetic resonance imaging studies without evidence of any VHL-associated lesions. There is limited prenatal, birth, and postnatal history available as she was adopted during infancy, and presented with early onset global developmental delay,undergoing interventional therapies during childhood. She was diagnosed with autism spectrum disorder and depression. To rule out underlying genetic etiology of global developmental delay, we performed chromosomal microarray and Fragile-X syndrome analysis. Her chromosomal microarray was normal whereas Fragile- X syndrome analysis incidentally revealed a premutation carrier status with 30 and 57 CGG repeats in the *FMR1* gene. The patient and her family were provided comprehensive genetic counseling about these results.

## Discussion

Von Hippel-Lindau (VHL) is an autosomal dominant hereditary tumor predisposition syndrome caused due to a germline pathogenic variant in the *VHL* gene (4). VHL syndrome predisposes an individual to various tumors such as cerebellar and spinal hemangioblastomas, retinal angiomas, renal cell carcinoma, and pheochromocytoma (1, 5). Other tumors such as pancreatic neoplasms, pituitary hemangioblastomas, and duodenal carcinoid tumors have also been rarely reported. The disorder has a high disease penetrance that is estimated to be about 97% by the age of 60 years (6). Patients with VHL syndrome have a shortened life expectancy secondary to complications related to cerebellar hemangioblastoma, renal cell carcinoma, and pancreatic neoplasms (1). Although VHL-associated tumors usually manifest at a younger age compared to sporadic tumors (6), they appear to be more responsive to chemotherapeutics (7), and less aggressive with respect to their local recurrence and metastatic involvement (8). Present surveillance guidelines recommend age-based screening with dilated eye examination, plasma metanephrines, MRI of brain, spine, abdomen, and internal auditory canal.

**Figure 3:**
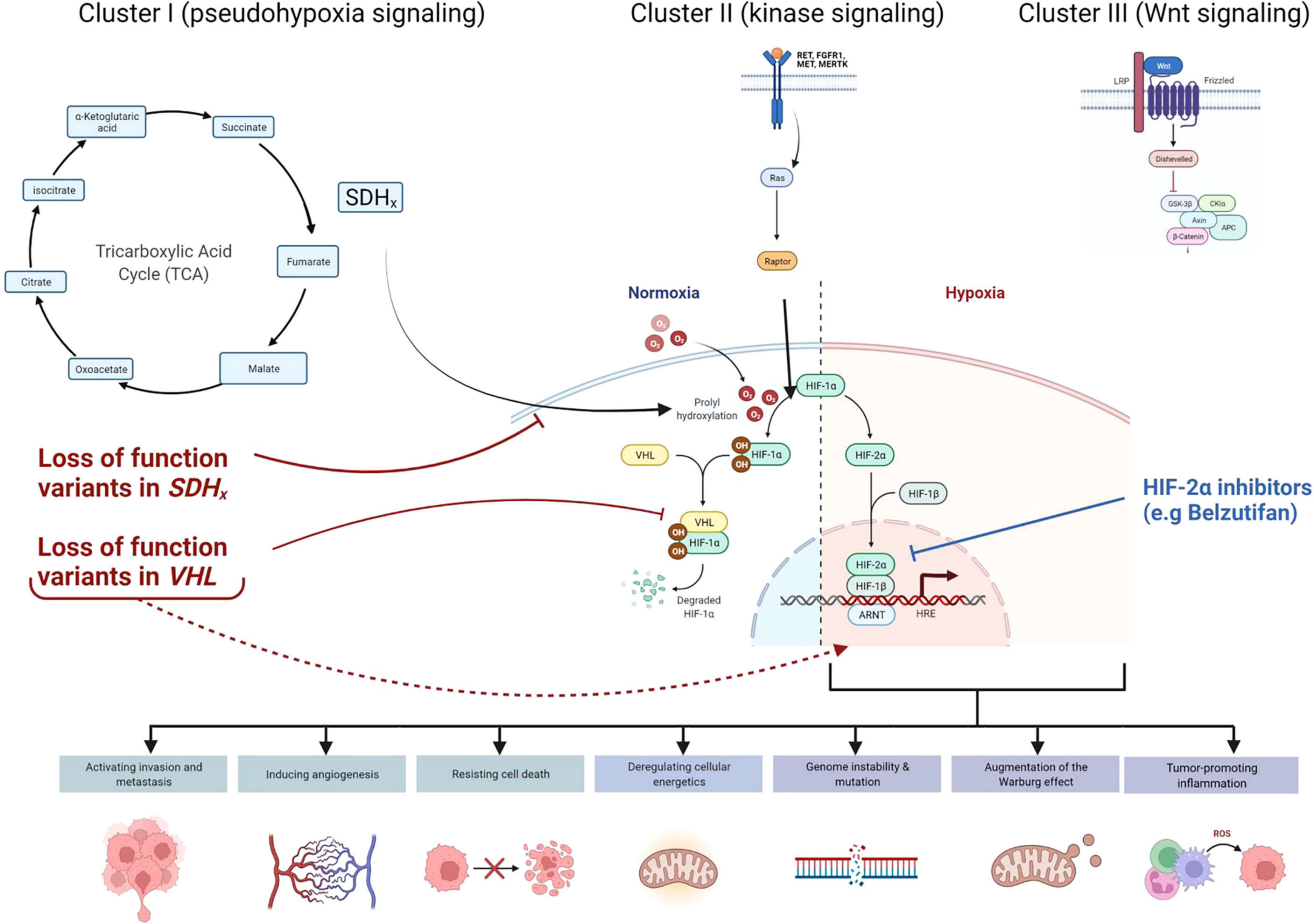
Cellular pathways and genes involved in hereditary paraganglioma-pheochromocytoma (PGL/PCC) syndrome and Von Hippel Lindau (VHL) disease. Approximately 40% of all cases of PGL/PCC cases are associated with germline pathogenic variants in the pseudo-hypoxic signaling pathway (Cluster I), kinase signaling (Cluster II), or Wingless and Int-1 (Wnt) signaling (Cluster III). Pathogenic variants in the *SDHx* genes (*SDHA, SDHB, SDHC*, and *SDHD*) and *VHL* gene are classified under the Cluster I genes. The SDH complex plays an important role in energy metabolism through the tricarboxylic acid (TCA) cycle. Both *VHL* and *SDHx* encode for proteins that target the protein HIF1-α for degradation. Pathogenic loss of function variants in *VHL* and *SDHx* genes leads to abnormal accumulation of HIF1-α and upregulation of downstream pathways, which results in increased expression of various proteins and augmentation of the Warburg effect. These negative effects may be mitigated by novel therapies such as HIF2-α inhibitors (e.g Belzutifan).

Collectively, hereditary paraganglioma-pheochromocytoma (PGL/PCC) syndromes are rare neuroendocrine tumors with an estimated incidence of approximately 2-8 cases per million per year (9). Approximately 40% of all cases of PGL/PCC are associated with germline pathogenic variants (2, 10, 11) in the pseudo-hypoxic signaling pathway (cluster I), kinase signaling (cluster II), or Wingless and Int-1 (Wnt) signaling group (cluster III) (**Figure 3**) (12). Pathogenic variants in the SDHx genes **(** *SDHA, SDHB, SDHC*, and *SDHD* **)** are classified under the cluster I genes which results in dysfunction of succinate dehydrogenase (SDH) leading to competitive inhibition of the enzyme, prolyl hydroxylase, involved in the degradation of hypoxia-inducible factor 1- α (HIF1- α) (2, 13, 14). SDHx-related PGL/PCC are relatively new tumor predisposition syndromes that include PGL and PCC, and rarely renal cell carcinoma, pituitary adenoma, gastrointestinal stromal tumors, and pancreatic neuroendocrine tumor (PNET) (15). Patients with PGL/PCC can manifest tumor at any age, although a majority would present between the third and fifth decade of life (16) with symptoms of excess catecholamine production, including hypertension, headache, diaphoresis, palpitations, and tremors. *SDHA* pathogenic germline variants are present in up to 30% of wild-type gastrointestinal stromal tumors and 10% of patients with PGL and PCC (Boikos et al, 2016). A recent study identified only 10 patients harboring a pathogenic germline *SDHA* variant in 4,974 pediatric and adult patients with a variety of solid tumors (17). Out of these 10 patients with underlying *SDHA* pathogenic variant, 2 had gastrointestinal stromal tumor, and the remaining had melanoma, neuroblastoma, breast, colon, renal, prostate, endometrial, and bladder cancer.

In contrast to *SDHB* which is a highly penetrant tumor predisposition gene, *SDHA* confers a much lower penetrance and severity, estimates of which are largely unknown (18, 19). Present surveillance guidelines for SDHx-associated PGL/PCC recommend plasma metanephrines, whole body and dedicated neck MRI, and complete blood count starting from age 6-8 years (19). Our patient represents the first case of a metastatic PNET in the setting of germline heterozygous pathogenic variants in the *VHL* and *SDHA* genes. PNETs are clinically heterogenous tumors originating from neuroendocrine cells of the pancreatic islets and usually follow a variable clinical course, with a low five-year survival rate in approximately 60% of patients (20). PNET occur in approximately 9 to 17% of patients with VHL disease (8) but have only been reported in one individual with a germline *SDHD* pathogenic variant (15). Since PNETs are seen in VHL and not observed so far in SDHA-related PGL/PCC syndrome, we postulate that the underlying genetic etiology would be the pathogenic variant with a second hit in the *VHL* gene. Although a second pathogenic variant was not reported by the tumor molecular testing, it is possible that the second hit could not be detected due to limitation of the tumor-based molecular analyses. These include possibility of somatic second hit being present in the promoter or deep enhancer region, or promoter methylation (21). Loss of heterozygosity (LOH) analysis could not be performedin the patient’s tumor type, as the laboratory only performs LOH analysis in ovarian tumors

Multi-locus inherited neoplasia allele syndrome (MINAS) is a relatively new entity that was initially reported in *BRCA1*/*BRCA2*-related cancers (21). With increasing adoption of next generation sequencing technologies for cancer susceptibility germline (CSG) testing, there has been a rise in reports of non-*BRCA1*/*BRCA2* related MINAS. A recent study by McGuigan et al. reported that atypical tumor phenotypes comprised of about 15% of non-*BRCA1*/*BRCA2* MINAS cases, which could be secondary to complex interactions between the relevant CSGs (21).

Interestingly, on a molecular level, both *VHL* and *SDHA* encode for proteins that target the protein HIF-α for degradation (22). Absence of these proteins would lead to abnormal accumulation and upregulation of HIF, resulting in increased expression of various proteins (e.g vascular endothelial growth factor, platelet-derived growth factor, matrix metalloproteinases and transforming growth factor-alpha), and augmentation of the Warburg effect by HIF-α. The Warburg effect relates to the phenomenon seen in tumor cells that effectively promotes growth and development. Even in normoxia, there is a metabolic shift in which tumor cells preferentially use glycolysis to generate adenosine triphosphate rather than the tricarboxylic acid (TCA) cycle (14). The SDH complex plays an important role in energy metabolism through the TCA cycle, and the reduction of ubiquinone to ubiquinol *via* the electron transport chain. Therefore, pathogenic variants in the genes involved in the SDH complex could result in accumulation of the ‘oncometabolite’ succinate, leading to disruption of the succinate to fumarate ratio that would inhibit the enzymatic degradation of HIF-α. Accumulation of succinate may also destabilize the redox state and cause mitochondrial dysfunction by increasing reactive oxygen species production and utilizing glutamine as an energy source (14). Epigenetic dysregulation has also been implicated in the malignant potential of SDHx-mutated PGL/PCC (3) and may be a contributing factor in the metastatic presentation of our patient’s PNET at diagnosis. As both these genes converge on the pseudo-hypoxia signaling pathway, we hypothesize that the co-occurrence of germline pathogenic variants in both the *VHL* and *SDHA* genes could have an impact on the long-term prognosis. Although our hypothesis is limited by the lack of functional studies and a single case report, further studies looking at this genotype-phenotype correlation in similar cases will be helpful. Co-occurrence of two germline variants in *VHL* and *SDHA* in our patient also creates an opportunity for the use of novel therapeutics based on the convergence of these two genes within the pseudo-hypoxia signaling pathway. Recently approved selective HIF inhibitors could be one of the therapeutic considerations in our patient as it has been shown to be effective in patients with renal cell carcinoma due to an underlying germline *VHL* pathogenic variant (23). HIF-2α overexpression is documented in VHL disease associated renal cell carcinoma (24). Preclinical studies indicated the potential efficacy of HIF-2α subunit inhibitors, which blocks the HIF pathway proximally and limits tumor growth in clear cell renal cell carcinoma (25, 26). Subsequent clinical trials documented the benefits and safety of HIF-2α inhibitors (e.g belzutifan) in both sporadic (27) and VHL-disease associated renal cell carcinoma (23). Belzutifan was also efficacious in reducing tumor size of non-renal cell carcinomas in VHL patients, including pancreatic neuroendocrine tumors, central nervous system hemangioblastomas and retinal hemangioblastomas (23). The U.S Food & Drug Administration (FDA) approved Belzutifan for renal cell carcinoma and non-renal cell neoplasms associated with VHL disease in August 2021 and remains a promising therapy for our patient who presented with both metastatic pancreatic neuroendocrine tumor as well as retinal hemangioma.

## Conclusion

The co-occurrence of *VHL* and *SDHA* pathogenic variants implicated in PNET is described in this case report. It opens the way for additional exploratory studies to determine the significance of co-occurrence of these variants in terms of tumor penetrance, severity, and outcomes, as well as for the development of novel therapeutic approaches targeting the shared cellular pathway involved in VHL and SDHA-related etiopathogenesis.

## Data Availability Statement

The original contributions presented in the study are included in the article/supplementary material. Further inquiries can be directed to the corresponding author.

## Ethics Statement

Ethical review and approval was not required for the study on human participants in accordance with the local legislation and institutional requirements. The patients/participants provided their written informed consent to participate in this study.

## Author Contributions

MT and BC drafted the original manuscript and created the figure, KD obtained consent and performed counseling for the testing coordinated in the patient. MG provided the pathology images and drafted the pathology description. TS provided radiology images and drafted the imaging description. AS provided patient information and performed manuscript revisions. All authors contributed to the article and approved the submitted version.

## Conflict of Interest

The authors declare that the research was conducted in the absence of any commercial or financial relationships that could be construed as a potential conflict of interest.

## Publisher’s Note

All claims expressed in this article are solely those of the authors and do not necessarily represent those of their affiliated organizations, or those of the publisher, the editors and the reviewers. Any product that may be evaluated in this article, or claim that may be made by its manufacturer, is not guaranteed or endorsed by the publisher.
